# Aligning early childhood science teaching beliefs, practices, and children’s learning outcomes: the impact of a professional development program

**DOI:** 10.3389/fpsyg.2025.1580018

**Published:** 2025-04-16

**Authors:** Shiyi Chen, Rebecca Sermeno, Kathryn Nikki Hodge, Rachel Louise Geesa, Hyuksoon S. Song, Burcu Izci, Zoe Froh, Sydney Murphy

**Affiliations:** ^1^Margaret Ritchie School of Family and Consumer Sciences, University of Idaho, Moscow, ID, United States; ^2^Department of Educational Leadership, Ball State University, Muncie, IN, United States; ^3^School of Arts, Sciences, Education, Georgian Court University, Lakewood, NJ, United States; ^4^College of Social Work, Florida State University, Tallahassee, FL, United States

**Keywords:** science teaching belief-practice alignment, young children, early childhood science education, professional development, science process skills

## Abstract

**Introduction:**

Science teaching belief-practice alignment is critical for ensuring high-quality, effective instruction that supports young children’s learning and engagement in science. The goal of this study is to examine the alignment between preschool and kindergarten teachers’ self-reported science teaching beliefs and observed practices before and after a science teaching professional development (PD) program and to explore whether the alignment is associated with children’s science learning outcomes. The research questions are: (1) Whether and to what extent do science teaching beliefs and practices align before and after the PD program? (2) Whether and to what extent are science teaching beliefs associated with children’s inquiry skills before and after the PD program?

**Methods:**

A total of 22 preschool and kindergarten teachers and 159 children (*M_age_* = 47.78 months) from a Northwest state in the U.S. participated in a science teaching PD program. Teachers’ self-reported science teaching efficacy and observed teaching practices and children’s directly assessed science inquiry skills were measured before and after a one-year science PD program.

**Results:**

Multilevel regression results revealed that science teaching beliefs were not correlated with practice before the PD but were significantly aligned following its completion. Further, while teachers’ science teaching beliefs were not associated with children’s inquiry skills before the PD, a positive correlation emerged after the PD.

**Discussion:**

Our findings suggest the potential of targeted PD programs in fostering early childhood teachers’ science teaching belief-practice alignment. The results also indicate that teachers’ science teaching beliefs may not be a robust predictor of children’s science learning outcomes unless they are aligned with teaching practices.

## 1 Introduction

Teaching beliefs, such as attitudes and efficacy toward teaching a subject, play a critical role in shaping teachers’ instructional practices and children’s learning outcomes (Oppermann et al., 2019; Perera and John, 2020). When positive teaching beliefs align with evidence-based, effective teaching practices, it creates an environment conducive to learning (Haatainen and Aksela, 2021; Kabiri, 2023; Kehoe and McGinty, 2024). Teaching beliefs can serve as mental frameworks that guide instructional decision-making; however, teaching beliefs do not always align with teaching practice in the classroom (Chen et al., 2024a; Buehl and Beck, 2014; Vitiello et al., 2020).

This study adopts two theoretical frameworks – Theory of Planned Behavior (Ajzen, 1991) and Social Cognitive Theory (Bandura, 1986). Theory of Planned Behavior suggests that individuals’ behaviors are guided by their attitudes (beliefs about the behavior), subjective norms (social pressures), and perceived behavioral control (self-efficacy). Applied to early childhood science teaching, teachers’ science teaching efficacy beliefs may represent their confidence in effectively delivering science instruction, which could impact their actual classroom practices. In turn, this alignment could influence children’s academic achievements (Fauth et al., 2019). Social Cognitive Theory (Bandura, 1986) emphasizes the dynamic interplay between personal factors (e.g., beliefs), behaviors (e.g., teaching practices), and environmental influences (e.g., professional development). The relation among these three factors is called Triadic Determinism (Woodcock and Tournaki, 2023). Central to Social Cognitive Theory is self-efficacy, which refers to one’s perceived competence in achieving certain outcomes (Schunk and DiBenedetto, 2020). Research shows that self-efficacy is related to motivation, task persistence, and overall performance (Schunk, 2023). In the context of this study, teachers’ science teaching beliefs reflect their self-efficacy, while their observed practices represent the enactment of these beliefs in classroom settings. We adopt Social Cognitive Theory as the second framework because it provides a lens for understanding how PD programs influence both science teaching self-efficacy and practices. This framework is particularly relevant for exploring how teaching belief-practice alignment impacts children’s learning, as it addresses the reciprocal interactions that shape educational outcomes.

An important form of teaching belief in science education is science teaching efficacy, which is a teacher’s belief in their ability to produce desired learning outcomes (Velthuis et al., 2014). When considering factors impacting science teaching efficacy, Chen et al. (2022) found the most influential factor on science teaching efficacy was mastery experiences (i.e., past successful science teaching experience). Teachers’ previous science learning, beliefs on the importance of science, and interpersonal support also play a role in shaping their science teaching efficacy (Chen et al., 2022). Teachers with higher science teaching efficacy tend to provide more science materials, engage more with science, and provide more frequent hands-on science activities (Bekirler and Siğirtmaç, 2024; Gerde et al., 2018). Furthermore, teachers’ science teaching efficacy is correlated with children’s science learning efficacy and motivation (Bekirler and Siğirtmaç, 2024; Oppermann et al., 2019). However, some researchers have reported that early childhood teachers’ teaching efficacy in science is significantly lower than their teaching efficacy in literacy and mathematics, and higher science teaching efficacy does not always align with science teaching instructional quality (Gerde et al., 2018), which is closely linked to children’s science conceptual learning and inquiry skills gain (Perera and John, 2020).

Children’s rudimentary conceptual understanding of science and science process skills begin to develop as early as infancy (Gopnik, 2020). These skills then undergo rapid transformation with maturation and early science learning experience (Adbo and Vidal Carulla, 2020). Due to their cognitive constraints, young children’s scientific understanding can be human-centered (Fusaro and Smith, 2018). For instance, a young child may believe that plants “eat” soil to grow, just as humans eat food, rather than understanding that plants absorb nutrients and water from the soil and produce energy through the process of photosynthesis. Teachers’ skillful, developmentally appropriate science teaching practices can steer children away from misunderstanding and catalyze children’s science learning (Ravanis, 2022). Previous research has shown that the quality of early childhood science teaching is a robust predictor of children’s scientific conceptual understanding, process skills gain, and later STEM achievements (Bustamante et al., 2023).

A key to early childhood science education is developmental appropriateness – science teaching and learning should be tailored to young children’s cognitive, social, emotional, and physical developmental levels (Piaget and Inhelder, 1969). In practice, early childhood science education heavily depends on hands-on activities, observation, exploration, and teachers’ guidance, such as modeling, asking questions, activating previous knowledge, and providing feedback (Studhalter et al., 2021). Quality early childhood science teaching is developmentally appropriate and inquiry-based; it nurtures young children’s natural curiosity about the world and fosters a positive attitude toward science learning (Larimore, 2020). Research also highlights the importance of meaningful and authentic science learning that is related to children’s daily experiences, allowing children to make connections between what they already know with the new knowledge (Ravanis, 2022). However, early childhood science practices often lag behind those in other domains, such as literacy and math. Gerde et al. (2018) found that, in their sample, 75–99% of early childhood teachers conducted literacy and mathematics classroom activities a week, but only 42% of the teachers engaged in science learning that often. The current early childhood science teaching practices are less than ideal. Based on a classroom observational study (Chen et al., 2024a), teachers sometimes miss opportunities to provide feedback on children’s misunderstandings during science activities. This could be due to limited teacher preparation and resources, indicating the need for targeted PD programs to strengthen this area (Méndez et al., 2017; Shirrell et al., 2019).

The alignment of early childhood science teaching beliefs and practices is important for promoting children’s science learning outcomes (Zee and Koomen, 2016). Teaching belief-practice alignment reflects the consistency between what teachers perceive as valuable instructional strategies and how they implement these strategies in the classroom (Chen et al., 2024b). Such alignment enhances the fidelity and intentionality of instructional practices, therefore providing children with quality learning experiences (Borg, 2017). On the other hand, misaligned beliefs and practices can hinder the effectiveness of instruction, as teachers may struggle to enact high-quality practices that they believe they are capable of implementing, leading to missed learning opportunities for children and work-related stress and anxiety for teachers (Martin et al., 2019; Vitiello et al., 2020). It is also important to note that belief-practice alignment alone is necessary but insufficient for producing effective instruction; the quality of teachers’ beliefs (e.g., whether they match content knowledge) is important as well (Gess-Newsome et al., 2019). Teachers’ science teaching beliefs tend to positively impact students’ outcomes when teachers’ beliefs are grounded in research and effectively translated into instructional practices (Piyakun and Phusee-Orn, 2025).

Teaching belief-practice alignment is particularly important for early childhood science education due to the unique demands of inquiry learning in this developmental stage (Bjorklund, 2022). First, inquiry learning in early childhood relies heavily on experiential activities, where children develop inquiry skills not only by hands-on exploration but also by observing and modeling their teachers (Raven and Wenner, 2023). Therefore, it is essential for teachers to hold positive science teaching beliefs and to enact instructional practices that effectively demonstrate how to approach a problem, interpret data, and persevere through challenges (Wan et al., 2021). Second, due to their developing cognitive capacity (Bjorklund, 2022), young children tend to learn more from assisted discovery activities (i.e., a type of inquiry learning scaffolded by teachers) than by fully child-led exploration (Alfieri et al., 2011). Aligned teaching beliefs and practices enable teachers to provide consistent, intentional scaffolding, creating rich opportunities for children to engage meaningfully with scientific inquiry (Zee and Koomen, 2016).

PD programs have the potential to enhance early childhood teachers’ science teaching belief-practice alignment mainly by addressing the gaps in science pedagogical content knowledge, enhancing science teaching self-efficacy, offering hands-on practices, and providing constant feedback (Li et al., 2022). We argue that continuous feedback through ways such as coaching, journaling, and assessments could be the main mechanism through which teachers reflect on their teaching beliefs and reconcile them with evidence-based teaching practices. Additionally, PD programs often incorporate actionable instructional strategies such as modeling and asking guiding questions, which bridge the theoretical understanding of teaching with practical applications in the classroom (Fischer et al., 2018). As teachers develop a deeper understanding of how to implement science teaching strategies, they are more likely to align their beliefs with their observed practices, resulting in more consistent and effective instruction that supports children’s learning outcomes.

Current research on domain-specific teaching belief-practice alignment mostly focuses on literacy and mathematics; much less attention is paid to science education, especially during early childhood (Bereczki and Kárpáti, 2021; Mao and Crosthwaite, 2019; Sengul et al., 2020). Moreover, much of the existing work on belief-practice alignment adopted teachers’ self-reported teaching practices, which could be subject to social desirability and might not accurately reflect the actual instructional practices in the classroom (Capps et al., 2016; Gibbons et al., 2018; Yurekli et al., 2020). Also, few studies have explored how belief-practice alignment may impact children’s learning outcomes, such as inquiry skills (Fauth et al., 2019; Perera and John, 2020). Additionally, the role of external factors, such as PD programs, in fostering belief-practice alignment remains underexplored (Fischer et al., 2018; Mansour, 2013), leaving questions about how to best support teachers in integrating beliefs and practices effectively. To address these empirical gaps, the present study aims to explore early childhood science teaching beliefs’ alignment with observed practices and their association with children’s learning outcomes. We ask the following research questions:

*RQ1*: Whether and to what extent do science teaching beliefs and practices align before and after the PD program?

*RQ2*: Whether and to what extent are science teaching beliefs associated with children's inquiry skills before and after the PD program?

## 2 Materials and methods

The present study adopts a within-subject quasi-experimental design. We have selected this research design for two reasons. First, the within-subject design allows us to control individual variabilities, such as teaching experience, teacher training, and baseline beliefs. This design strengthens the validity of this study, ensuring that the changes in teachers’ and children’s outcomes are more likely due to the PD program rather than pre-existing group differences (Montoya, 2023). Second, we did not include a control group or use random sampling because of the challenges of recruiting participants in a rural area with very low population density. To address these constraints, we employed a within-subjects quasi-experimental design to ensure that our analysis had sufficient statistical power (Gopalan et al., 2020).

### 2.1 Participants and settings

This study was approved by the Institutional Review Board (IRB) at the lead author’s university (IRB protocol code 21–233). Eligible participants were preschool and kindergarten teachers and children (age = 4–6 years, typically developing) within 2 h’ driving distance from the lead author’s university from rural regions of a northwest state in the U.S. Participants were recruited using a convenient sampling method. Trained research assistants contacted potential participating teachers via phone calls, emails, and a recruitment event at a regional child development conference in the summer of 2023. Participating teachers then distributed parental consent forms to the families of eligible children in their classrooms and collected the signed forms from parents. For each teacher, approximately six children were randomly selected by the research assistants for data collection from all the children with parental consent. Consent forms, along with other identifiable paper data, were stored in a locked file cabinet in the lead authors’ office. Digital data were stored in a password-protected laptop computer. Only deidentified data were used for data analysis and reporting.

A total of 25 teachers consented, and 22 of them completed both pre and posttest data collection (*N_teacher_* = 22) (Table 1). On average, the teachers’ age was 37.10 years old (*SD* = 10.97, range = 20–56), they were predominately White (68%), 50% had a Bachelor’s degree and above, and their teaching experiences ranged from 0.25 to 31 years (*SD* = 6.69). Out of the 16 teachers who reported their degree majors, eight majored in Early Childhood Development and Education, two majored in Elementary Education, and six majored in non-education related areas, such as Communications, Forestry, Accounting, and Animal Science. The child sample consisted of 159 children and had slightly more girls than boys (*N_girl_* = 81, *N_boy_* = 78), with an average age of 47.78 months (SD = 8.45, range = 32–72). A *post-hoc* power analysis showed that our sample size enabled the analysis to reach a statistical power of 0.83 with an alpha of 0.05.

**Table 1:** Participants’ demographic information.

		*N*	M/percent	SD	Min.	Max.
Teacher						
Gender	Female	22	100			
Age (years)		19	37.10	10.97	20	56
Ethnicity/Race	Hispanic	3	14			
	None-Hispanic White	15	68			
	Other	4	18			
Grade	Preschool	21	95			
	Kindergarten	1	5			
Have a certification		6	27			
Have a CDA		13	59			
Degree	GED	3	14			
	HS	4	18			
	AA	4	18			
	BA/BS	9	41			
	MA/MS	2	9			
Experience (years)		20	9.35	6.69	0.25	31
Child						
Gender	Boys	78	49			
	Girls	81	51			
Age (mo.)		159	47.78	8.45	32	72
Ethnicity/Race	Hispanic	14	3			
	Non-Hispanic White	131	82			
	American Indian	5	3			
	Black	2	1			
	Bi-or Multi-racial	7	11			

AA, Associate degree; BA/BS, Bachelor’s degree; CDA, Child Development Associate Credential; MA/MS, Master’s degree; mo, month; yrs, years.

### 2.2 Professional development program design

**Figure 1:**
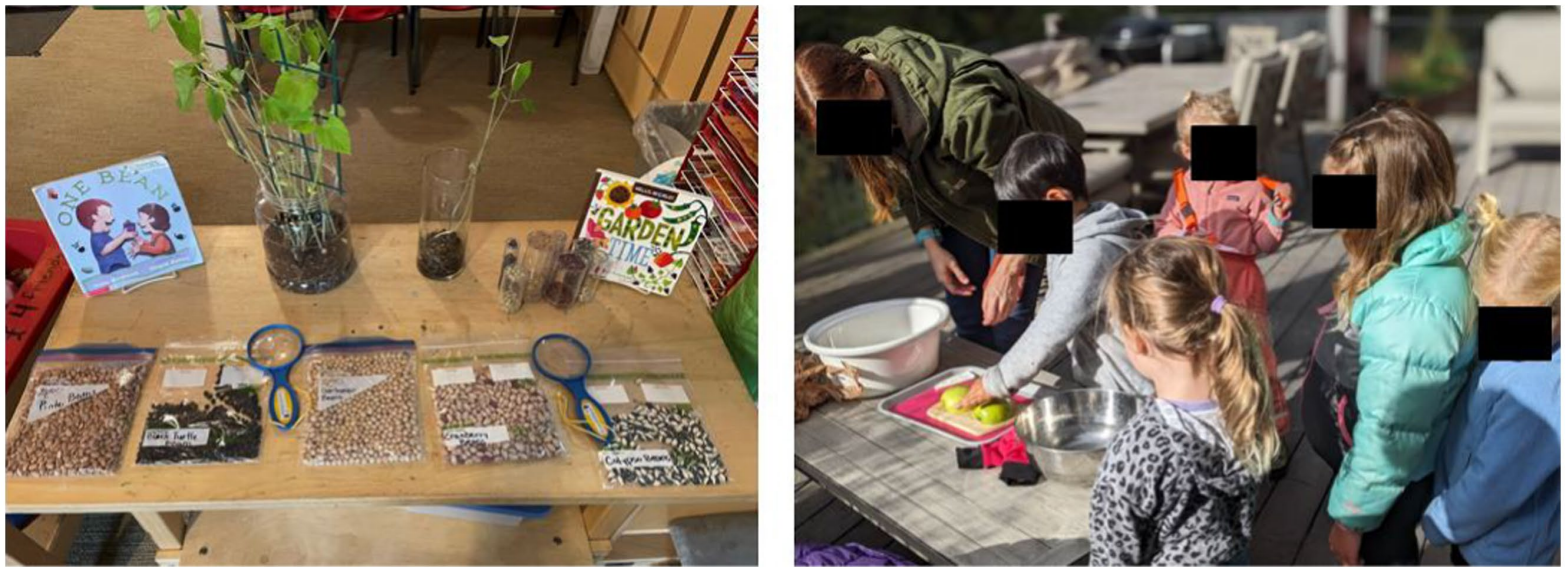
Photographs of the PD program implementation. Left: bean exploration; right: sink or float experiment with apples and pears.

The present study is part of a larger PD project – Farm to Early Care and Education (Chen et al., 2024c). Data presented in this manuscript are from the second year of this PD program, from September 2023 to May 2024. This experiential PD program aims to improve rural preschool and kindergarten teachers’ science teaching capacity using fruit-and vegetable-themed curricula that align with the rural farming culture. In this nine-month PD program, teachers received monthly deliveries of a “Harvest of the Month kit” that included print copies of the monthly curriculum, science experiment materials (e.g., cups, seeds), and arts and crafts materials, local farm produce, children’s books, and children’s observation journals. Each month’s curriculum included four weekly lesson plans featuring a different local vegetable, fruit, or grain (e.g., lentils, peaches, and tomatoes). The curriculum focuses on developmentally appropriate science teaching strategies and scientific concepts such as plants’ life cycles, germination, and plant parts (see Figure 1). Also, teachers were asked to complete a monthly training module before implementing the curriculum. The monthly training took place at the beginning of each month on Canvas – a virtual learning platform, where teachers reviewed videos explaining the curriculum content and teaching strategies and videos that demonstrated the real-life enactment of those teaching strategies. The online training module also included teaching resources such as printable materials, songs, dances, and recipes.

### 2.3 Data collection procedure

Data in the present study were collected between fall 2023 and spring 2024. Upon receiving the teachers’ consent forms and the children’s parental consent forms, teachers were given an online survey link to complete self-reported demographic questionnaires and a validated rating scale assessing their science teaching beliefs. Teachers completed the same science teaching efficacy scales upon exiting the program. Survey data were exported from the online survey software Qualtrics and cleaned for data analysis. To assess teachers’ science teaching practices, trained research assistants scheduled two in-class observations with each teacher in fall 2023 and spring 2024. Teachers were notified of the observation at least 2 weeks in advance to ensure they had sufficient time to prepare the lessons. Teachers’ science teaching practices were evaluated using a validated tool, Dimension of Success (see the next section for a detailed description of this tool). On the observation day, the research assistants sat in a corner of the classroom, observed teachers’ science teaching practices (each observation was about 15 min on average), and took detailed field notes. The field notes were scored by the same research assistants who observed the teachers’ teaching within a week of the observation.

### 2.4 Measurements

#### 2.4.1 Teachers’ science teaching efficacy

Teachers’ science teaching efficacy was measured by the Science Teaching Efficacy and Beliefs (STEB; *α _STEB_* = 0.90) and Science Teaching Outcome Expectancy (STOE; *α _STOE_* = 0.93) subscales in the Elementary Teacher Efficacy and Attitudes toward STEM Surveys (T-STEM; Friday Institute for Educational Innovation, 2012) (Appendix A). STEB and STOE are five-point Likert scales, including 20 items in total. An example of the STEB scale includes: “When a student has difficulty understanding science concept, I am confident that I know how to help the student understand it better.” An example of the STOE scale includes: “Students’ learning in science is directly related to their teacher’s effectiveness in science teaching.” Composite scores were created for STEB and STOE by aggregating the rating of each item under each subscale.

#### 2.4.2 Teachers’ science teaching practices

The observational tool, Dimensions of Success (DoS; Shah et al., 2018) (Appendix B) was used to assess the quality of teachers’ science teaching practices. Trained research assistants observed each teacher’s science teaching practices in their classes and took detailed notes during the PD program in October 2023 and April 2024. Observation field notes were scored across 12 dimensions on a 4-point ranging from “Evidence Absent (1)” to “Compelling Evidence (4).” The 12 DoS dimensions (*α* ranges from 0.79–0.90) are under four broad domains: Features of the Learning Environment (includes Organization, Materials, and Space Utilization), Activity Engagement (including Participation, Purposeful Activities, and Engagement with STEM), STEM Knowledge and Practices (including STEM Content Learning, Inquiry, Reflection), and Youth Development in STEM (including Relationships, Relevance, and Youth Voice). We created composite scores for each teacher by aggregating scores across 12 dimensions.

#### 2.4.3 Children’ science inquiry skills

Children’s science inquiry skills were measured by the Scientific Inquiry Processes subset in the Science Learning Assessment (SLA) (Samarapungavan et al., 2009) (Appendix C). The Scientific Inquiry Processes subset (*α* = 0.87) included nine items that tap into three core elements of scientific inquiry: (1) Science is a process of inquiry based on asking questions and making predictions about the natural world, (2) The empirical basis of science: Scientific ideas are evaluated by their correspondence or fit to empirical evidence, and (3) Scientists use simple tools to gather, record, analyze, and share data. Question prompts were read to the child by their classroom teacher in a one-on-one setting in a quiet corner of the classroom. Children will then verbalize the answer or point to one of the picture options they think is correct. There are eight multiple-choice questions and one short-answer question. For example, item number four shows a red ball and three girls and the prompt reads “One of these girls makes a prediction about the ball: Which one?” Children are then asked to choose one of the girls’ responses (also read to children) shown in the picture: Girl A – “The ball can bounce;” girl B – “The ball is red;” girl C – “My dress is green.” Children’s responses were scored by two trained research assistants independently as correct (1) and incorrect (0). Scoring differences were discussed with the lead author and resolved. We created composite scores for each child by aggregating scores of nine items.

### 2.5 Data analysis

A multiple regression analysis was conducted to examine the relationship between teachers’ science teaching beliefs and their observed teaching practices. The dependent variable was teaching practices, measured through classroom observations. The independent variable was teaching beliefs, assessed via self-reported efficacy scores. Teachers’ level of education was included as a covariate to control for its potential influence on teaching practices. We first conducted preliminary data analysis, such as checks for multicollinearity, residual normality, and missing data (Little and Rubin, 2019; Osborne and Waters, 2002).

To evaluate the relationship between teachers’ science teaching practices and children’s inquiry skills, a multilevel modeling (MLM) approach was employed (Hox, 1998). This method was selected to account for the nested structure of the data, where children (Level 1) were nested within classrooms/teachers (Level 2). MLM allows for the separation of variance attributable to individual child-level differences from variance explained by teacher-level factors, thereby providing accurate parameter estimates while considering the hierarchical data structure (Peugh, 2010).

Unconditional models were fitted first to estimate the intraclass correlation coefficient (ICC), quantifying the proportion of variance in children’s inquiry skills attributable to differences between classrooms/teachers. Teaching practices were added as a Level 2 predictor to examine their direct effect on children’s inquiry skills, controlling for baseline inquiry skills where applicable. Random intercepts were included to allow for classroom-specific deviations in inquiry skills, capturing unobserved heterogeneity at the classroom/teacher level (Muthén and Asparouhov, 2009).

## 3 Results

### 3.1 Preliminary analysis

For preliminary data analysis, we examined descriptive statistics, data normality, bivariate correlation, and Little’s MCAR test of missing data (Little and Rubin, 2019). Results showed that data were normally distributed, and data were missing at random. Three possible outliers were within three standard deviations of the mean, so we did not remove them (Osborne and Overbay, 2004). Regression assumption testing results showed that the data met the assumption for multiple regression and multilevel regression analyses.

### 3.2 Research question 1

To answer RQ1, *Whether and to what extent do science teaching beliefs and practices align before and after the PD program*?, we conducted two multiple regression analyses with the pretest and posttest belief scores as the independent variable in each model, respectively (Freund et al., 2006). The average teacher practice scores (i.e., time 1 scores plus time 2 scores divided by 2) were the dependent variables and teachers’ education levels were covariate in both multiple regression models. We used the average Dimensions of Success (DoS) scores to better represent each teacher’s science teaching practices. Results showed that, prior to participating in the science teaching PD program, both STEB and STOE were not correlated with the quality of teachers’ actual science teaching practice in the classroom, controlling for teachers’ education levels. After the PD program, teachers’ STEB was significantly and moderately correlated with the quality of their science teaching practices (*β_STEB_* = 0.44, *p_STEB_* < 0.001), controlling for their education levels. Teachers’ STOE scores were marginally significantly related to the quality of teachers’ science teaching practices (*β_STOE_* = 0.18, *p_STOE_* = 0.054). The results of RQ1 suggested that early childhood teachers’ science teaching beliefs, represented by STEB and STOE, were not aligned with their actual teaching practices in the classroom. However, the science teaching beliefs and practices alignment improved significantly after the PD program.

### 3.3 Research question 2

To answer RQ 2, *Whether and to what extent are science teaching beliefs associated with children’s inquiry skills before and after the PD program*?, we conducted multilevel regression modeling (Peugh, 2010). Given the nested structure of our data (i.e., children are clustered within classrooms/teachers), the assumption of independent observations in traditional regression analysis would be violated (Osborne and Waters, 2002). Specifically, children’s science learning outcomes may be influenced by shared experiences within the same classroom, such as the way their teacher delivers instruction. Therefore, we used a multilevel modeling approach to account for variance at both child level (Level 1), and classroom level (Level 2). Unconditional models were tested first without adding any predictors. Intra-class correlations (ICCs) showed that 21% of the variance in children’s inquiry skills at pretest and 24% of the variance at posttest can be attributed to differences between classes/teachers, justifying the use of multilevel modeling.

Two multilevel regression models were run with children’s science inquiry skills at pretest and posttest as the outcomes, respectively. Teacher pretest and posttest science teaching self-efficacy were added to each model as the predictor; teachers’ education levels and children’s age (in months) were used as the covariates in both models. Results suggested that, before participating in the PD program, teachers’ science teaching beliefs, both STEB and STOE, were not significantly related to children’s scientific inquiry skills (95% *CI_STEB_* = [−0.34, 1.45], *p_STEB_* = 0.22; *CI_STOE_* = [−2.08, 2.50], *p_STOE_* = 0.86), controlling for teachers’ education level and children’s age. This suggested that early childhood teachers’ self-reported science teaching beliefs were not a robust predictor of children’s learning outcomes in the absence of targeted intervention. Following the PD, a significant association between teachers’ STEB and children’s science inquiry skills was found (*β_STEB_* = 2.27, *CI_STEB_* = [0.69, 3.85], *p_STEB_* < 0.01). However, teachers’ STOE was not related to children’s scientific inquiry skills (*CI_STOE_* = [−0.63, 5.02], *p_STOE_* = 0.13). This result indicated that teachers’ self-reported science teaching efficacy beliefs, not outcome expectancy, were correlated with children’s scientific inquiry skills after targeted science teaching PD training.

## 4 Discussion

The present study examined the alignment of early childhood teachers’ science teaching beliefs with their instructional practices and the association with children’s science inquiry skills before and after a professional development (PD) program. Data analysis results revealed that teachers’ self-reported science teaching beliefs were inconsistent with observed teaching practice in the classroom and were unrelated to children’s scientific inquiry skills prior to the PD program. However, the belief-practice alignment and beliefs’ correlation with children’s scientific inquiry skills significantly improved after the PD program.

### 4.1 Belief-practice alignment

Contrary to the Theory of Planned Behavior (Ajzen, 1991), results showed that before the PD program, there was no significant correlation between teachers’ science teaching beliefs [both self-efficacy beliefs (STEB) and outcome expectancy beliefs (STOE)] and their observed teaching practices. This finding suggests that teachers might have been engaging in science teaching practices that were inconsistent with their beliefs before the PD program, which could be due to contextual barriers such as lack of training, time constraints, or limited access to resources (Hayes et al., 2024), or self-reported beliefs’ social desirability bias (Dodou and de Winter, 2014). Our findings seem to be consistent with research on teaching beliefs’ alignment with observed classroom instructional practices (Gibbons et al., 2018; Kruse et al., 2021; Poulou et al., 2019). However, they contradict research on teaching beliefs’ alignment with self-reported teaching practices (Deehan and MacDonald, 2024; Dilekli and Tezci, 2016; Perera and John, 2020). While teachers may perceive their instructional practices as consistent with their efficacy beliefs, relying solely on self-reported instructional practice could be misleading (Buehl and Beck, 2014). As our results pointed out, teachers’ teaching beliefs did not match their enacted practices. Therefore, incorporating observational measures of actual teaching practices offers a more robust and objective assessment of belief-practice alignment. This is especially important for researchers using cross-sectional data as compared to longitudinal and PD-focused studies.

Following the PD program, teachers’ beliefs became significantly aligned with their practices. Specifically, teachers’ STEB moderately correlated with their teaching practices, and their STOE scores were marginally correlated with teaching practices. This improvement suggests that belief-practice alignment is not static but can be shaped through targeted interventions such as PD programs (Baez-Hernandez, 2019). We speculate that our PD improved early childhood teachers’ science teaching belief-practice alignment in several ways. First, our experiential curriculum and training provided teachers with basic plant and food science knowledge and developmentally appropriate teaching strategies, which empowered teachers to modify their instructional approaches to better reflect their beliefs about how children learn, and strengthened both their teaching efficacy and teaching practices (Leuchter et al., 2020). Second, the requirement for teachers to submit quarterly journal entries encouraged them to reflect critically on the highlights and challenges of their recent science teaching experiences. This reflective practice may have supported teachers in identifying discrepancies between their instructional beliefs and actual practices, prompting adjustments informed by deliberate self-evaluations (Vujnovic and Medic, 2022). Third, after each observation, trained RAs offered constructive feedback to teachers, which helped teachers refine their practices to better match their beliefs about effective teaching and learning. By fostering a continuous cycle of reflection and adaptation, the journaling process combined with coaching feedback likely enhanced teachers’ metacognitive awareness in their teaching practices in our study (Desimone and Pak, 2017). This dual approach of self-and objective evaluation encouraged teachers to actively monitor their instructional beliefs, assess their alignment with classroom practices, and make necessary adjustments based on children’s learning (Gardner-Neblett et al., 2021).

### 4.2 Beliefs’ association with children’s learning outcomes

Before the PD program, neither teachers’ STEB nor STOE in our study were significantly related to children’s learning outcomes measured as scientific inquiry skills, suggesting that teachers’ beliefs did not play a role in young children’s learning outcomes (Zee and Koomen, 2016). This finding is not surprising given that teachers’ science teaching beliefs are inconsistent with their actual classroom instructional practice in our study. As Perera and John (2020) pointed out, the possible correlation between teaching efficacy belief and students’ outcomes might be mediated by classroom instructional practices, which have a direct impact on students’ learning. A plausible explanation of our finding is that, before participating in the PD program, teachers may hold positive self-efficacy and outcome beliefs, but struggle to operationalize them effectively in their instruction due to reasons such as limited training and resources, therefore, limiting beliefs’ impact on student learning (Yurekli et al., 2020).

Following the PD program, a different pattern emerged. Teachers’ STEB scores were significantly associated with children’s scientific inquiry skills at posttest, indicating that increased teacher confidence in their ability to teach science effectively translated into improved learning outcomes. Given the alignment of teachers’ beliefs and practices after the PD program, it is likely that the training enhanced both their understanding of their science teaching beliefs and their ability to enact these beliefs in practice. This improved alignment may have empowered teachers to translate the strategies and knowledge gained during the PD into more effective instructional practices, which have a direct impact on children’s learning outcomes (Gess-Newsome et al., 2019). However, STOE scores remained unrelated to children’s inquiry skills, suggesting that outcome expectancy beliefs may play a different role than efficacy beliefs in classroom dynamics and outcomes (Chen et al., 2024b), which requires further exploration.

### 4.3 The role of PD in shaping science teaching beliefs’ alignment with practices and children’s outcomes

Our findings highlight the transformative role of PD programs in fostering belief-practice alignment and enhancing the influence of teacher beliefs on children’s outcomes. Prior to the intervention, teachers’ beliefs and practices operated independently, and beliefs were not associated with children’s learning outcomes. However, the PD program appeared to act as a catalyst, enabling teachers to become more adept at consciously integrating their beliefs into their teaching while refining their methods to better support children’s learning (Gardner-Neblett et al., 2021).

The effect of PD on teachers’ belief-practice alignment could be explained by Social Cognitive Theory (Bandura, 1986), which states that behavior is shaped by the dynamic interplay of personal factors (e.g., teaching efficacy beliefs), behavioral factors (e.g., teaching practices), and environmental factors (e.g., PD training). In this context, PD programs act as a key environmental influence, providing teachers with background knowledge and teaching strategies to bridge the gap between their beliefs and practices (Leuchter et al., 2020). Simultaneously, enhanced teaching efficacy beliefs (personal factors) may motivate teachers to implement effective teaching practices (behavioral factors) (Kabiri, 2023). This process reflects the theory’s principle of Reciprocal Determinism, wherein personal, behavioral, and environmental factors mutually influence each other (Bandura, 1986). Thus, PD does not simply influence teaching beliefs or practices in isolation but fosters a continuous feedback loop that aligns teachers’ internal beliefs with their external instructional practices, leading to improved coherence and effectiveness in their teaching (Baez-Hernandez, 2019). When teachers’ beliefs about teaching align with their practices, teachers may be more metacognitively aware of their attitudes and pedagogical content knowledge, and fine-tune their teaching practices as a result of the alignment (Buehl and Beck, 2014). It is worthwhile for future researchers to investigate the mechanisms through which teaching belief-practice alignment impacts children’s learning, specifically the role of adaptive teaching strategies, self-monitoring during teaching, and differentiated instruction.

Our study has important implications for the design of PD programs in early childhood education. Effective PD should prioritize not only enhancing teachers’ pedagogical content knowledge, but also providing practical support for translating beliefs into actionable teaching practices (Chen et al., 2024a). In particular, our study highlighted the importance of regular evaluation and feedback in a PD program – subjective and/or objective evaluation helps teachers construct a more well-rounded understanding of their teaching beliefs, instructional practices, and the connection between the two. Further, the distinct roles of STEB and STOE in children’s learning outcomes post-PD suggest that PD programs may need to purposefully address STOE. For example, enhancing STOE may require PD programs to explicitly focus on reinforcing teachers’ confidence in the tangible impact of their teaching on children’s learning outcomes (Sharp et al., 2022). This could be achieved by providing evidence-based examples of effective science teaching translating into measurable student progress, as well as creating opportunities for teachers to witness and reflect on the positive effects of their instruction to strengthen their belief in the impact of their teaching (Abrams et al., 2016). At the same time, we must acknowledge that contextual factors unrelated to PD programs, such as organizational support and classroom environments, may impact teaching beliefs and practices (Hayes et al., 2024). Future research should explore how these factors moderate the effect of PD programs on teaching beliefs and practices.

### 4.4 Limitations

Several limitations of this study should be considered when interpreting our findings. First, data were from rural areas in a northwest state in the U.S., which may limit the generalizability of the results to broader populations of early childhood educators and children (Schreier, 2018). Second, the observation data were collected at two time points during the PD, rather than before and after the PD program. The timing of the observation data collection allowed us to capture a more accurate and representative depiction of teachers’ science teaching practices. However, the lack of pretest and posttest observation data may limit the ability to draw definitive conclusions about longitudinal changes in belief-practice alignment due to the PD. Third, this study adopted a self-reported rating scale to measure science teaching efficacy. However, it may be subject to social desirability bias. Incorporating qualitative measures, such as interviews, could provide richer insights into belief-practice alignment (Zee and Koomen, 2016). Fourth, contextual factors that may impact our results, such as variations in the teacher-child ratio and teachers’ prior experience, were not controlled. Finally, teachers’ science teaching efficacy and children’s inquiry skills were measured immediately before and after the PD program. Without delayed posttest data, we could not determine whether the observed belief-practice alignment and its impact on children’s learning are sustained over time.

### 4.5 Future directions

Future studies could investigate the mechanisms through which PD program impacts teaching belief-practice alignment. For instance, research could examine whether enhanced pedagogical content knowledge, personalized feedback, or access to high-quality teaching resources mediates the relationship between PD participation and improved alignment. Such insights could inform effective PD program design. Moreover, future research should expand the scope of the present study by investigating the role of contextual factors, such as administrative support, work stress, curriculum and standards adoption, and resource availability, in moderating the relationship between belief-practice alignment and instructional quality. The results of such a study can provide valuable insights into how PD programs can be tailored to maximize their effectiveness. Additionally, future research should use a more representative sample and longitudinal data to produce results that can be generalized to a larger population.

## 5 Conclusion

This study addressed several empirical gaps, including the limited research on early childhood science education and the lack of research on the alignment of beliefs and practices in science teaching settings. The study’s strength lies in comparing self-reported beliefs with observed, actual classroom practices and adopting a within-subject design, which enables the examination of changes in belief-practice alignment over time and sheds light on a PD program’s impact on belief-practice alignment. Our results indicate that belief may not be a robust predictor of children’s learning outcomes unless it is consistent with actual instructional practices in the classroom. Therefore, researchers should exercise caution in relying solely on self-reported teaching beliefs as a predictor of students’ academic achievements. Our findings also highlight the importance of PD programs in bridging the gap between what teachers believe about science teaching and how they implement those beliefs in their classrooms. We encourage future researchers to further investigate the mechanism through which PD programs can improve science teaching-practice alignment.

Our study also has practical implications. First, it is important for teachers to reflect on their instructional beliefs and practices, fostering an understanding of how these elements interact. When teachers are aware of their beliefs and actively align them with their teaching in the classroom, they may be better equipped to create engaging, inquiry-based science learning environments that inspire curiosity and critical thinking in young learners. Also, our findings suggest that effective teaching is a dynamic interaction of personal, behavioral, and environmental factors. Education researchers, school administrators, and policymakers should create PD programs that offer opportunities for teachers to experiment, receive feedback, and collaborate with peers to ensure that training translates into meaningful classroom practices.

In conclusion, fostering belief-practice alignment is not merely a theoretical goal, but a practical strategy for improving teaching and learning. When teachers’ beliefs and practices are in harmony, a solid foundation for cultivating high-quality science learning experiences for children can be developed.

## Data Availability

The datasets are available upon request made to the corresponding author.
