# Supplementary material for: Aligning early childhood science teaching beliefs, practices, and children’s learning outcomes: the impact of a professional development program
Source: Front Psychol. 2025 Apr 16;16:1580018. doi: 10.3389/fpsyg.2025.1580018 (PMC12044615; doi:10.3389/fpsyg.2025.1580018)

Appendix A. Science Teaching Efficacy Beliefs and Outcome Expectancy

There are no right or wrong answers in this list of statements. It is simply a matter of what is true for you. Read every statement carefully and choose the one that best describes you.

	Strongly Disagree (1)	Disagree (2)	Neutral (3)	Agree (4)	Strongly Agree (5)
Science Teaching Efficacy Beliefs					
I am continually improving my science teaching practice.					
I know the steps necessary to teach science effectively.					
I am confident that I can explain to students why science experiments work.					
I am confident that I can teach science effectively.					
I wonder if I have the necessary skills to teach science.					
I understand science concepts well enough to be effective in teaching science.					
Given a choice, I would invite a colleague to evaluate my science teaching.					
I am confident that I can answer students' science questions.					
When a student has difficulty understanding a science concept, I am confident that I know how to help the student understand it better.					
When teaching science, I am confident enough to welcome student questions.					
I know what to do to increase student interest in science.					
Science Teaching Outcome Expectancy					
When a student does better than usual in science, it is often because the teacher exerted a little extra effort.					

The inadequacy of a student's science background can be overcome by good teaching.					
When a student's learning in science is greater than expected, it is most often due to their teacher having found a more effective teaching approach.					
The teacher is generally responsible for students' learning in science.					
If students' learning in science is less than expected, it is most likely due to ineffective science teaching.					
Students' learning in science is directly related to their teacher's effectiveness in science teaching.					
When a low achieving child progresses more than expected in science, it is usually due to the extra attention given by the teacher.					
If parents comment that their child is showing more interest in science at school, it is probably due to the performance of the child's teacher.					
Minimal student learning in science can generally be attributed to their teachers.					

Appendix B Dimension of Success

Note. Only part of the assessment is shown here due to copyright issues. Interested users can contact Partnerships in Education and Resilience https://www.pearinc.org/dimensions-of-success (Accessed on 25 March 2025)

Dimensions of Success
a pear observation tool
DoS

Part I: FIELD NOTES

(Type your field notes here; scroll down to enter ratings/evidence):

Instruction: Please fill out the "*Information*" section and use the "*Field Notes*" section to take notes on the classroom environment and teaching/learning activities <u>related to food & agriculture knowledge and/or science inquiry</u>, especially about the following:

- The physical environment and how it was used. Examples are, but are not limited to:
  - Science area (location, accessibility…).
  - Activity materials.
  - Classroom display (posters, artifacts…).
- Teacher's instruction. Examples are, but are not limited to:
  - Instructional strategies that teachers used during an activity (e.g., comparing, sorting, inquiry learning, predicting, discussion, problematizing).
  - Classroom engagement.
  - Instructional behaviors that support children's learning and understanding.
  - Teacher-child interaction (verbal and non-verbal).
  - Classroom management.

**Information**			
**Teacher ID**		**Date (mm/dd/yy)**	
**Teacher Name**		**Observer Name**	
**Site Name**		**Grade Level**	
**Curriculum month**		**Curriculum week**	
**Fruit/Veggie/Grain**		**Activity Name**	
**Start time**		**End time**	
**Field Notes**			

Appendix C. Scientific Inquiry Processes subset in the Science Learning Assessment

Note. Only part of the assessment is shown here due to copyright issues. Interested users can contact the authors (Samarapungavan et al., 2009).

This **one-on-one** assessment needs to be administered by the teacher to individual children in a **quiet** place. You will read the **blue text** word-for-word while pointing and showing the pictures to the child. Write down the child's answer in "*Child's response*: ___", under each question. You may repeat the question upon the child's request. Please do not prompt or give the answer to the child.

Two girls find a seed. The girl in green thinks it is a flower seed. The girl in blue thinks it is a tree seed. How can they find out what it is?

**Teacher: please write down children's response word-for-word.*

Child's response: ____

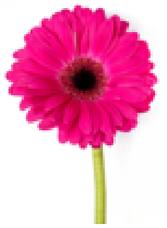

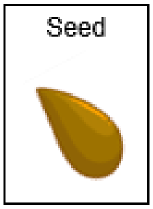

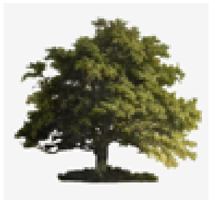

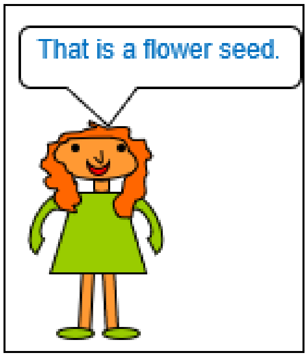

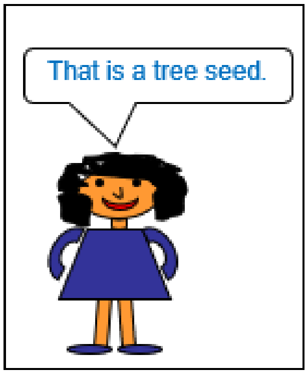

Supplement: Supplementary file 1 [file Data_Sheet_1.pdf]
